# Implementation and scalability of shared care models for chronic eye disease: a realist assessment informed by health system stakeholders in Finland, the United Kingdom, and Australia

**DOI:** 10.1038/s41433-023-02444-9

**Published:** 2023-03-06

**Authors:** Belinda Ford, Blake Angell, Hueiming Liu, Andrew White, Lisa Keay

**Affiliations:** 1 The George Institute for Global Health, Faculty of Medicine and Health, UNSW Sydney, 1 King Street Newtown, Sydney, NSW 2042 Australia; 2 Westmead Hospital Ophthalmology Department, Corner Hawkesbury and Darcy Rd Westmead, Sydney, NSW 2145 Australia; 3 Centre for Vision Research, Westmead Institute for Medical Research, Sydney Medical School, University of Sydney, Sydney, NSW Australia; 4 School of Optometry and Vision Science, Faculty of Medicine and Health, UNSW Sydney, Sydney, NSW 2052 Australia

**Keywords:** Health services, Eye diseases

## Abstract

**Background/Objectives:**

Several health systems have implemented innovative models of care which share the management of patients with chronic eye diseases between ophthalmologists and optometrists. These models have demonstrated positive outcomes for health systems including increased access for patients, service efficiency and cost-savings. This study aims to understand factors which support successful implementation and scalability of these models of care.

**Subjects/Methods:**

Semi-structured interviews were conducted with 21 key health system stakeholders (clinicians, managers, administrators, policy-makers) in Finland, United Kingdom and Australia between October 2018 and February 2020. Data were analyzed using a realist framework to identify the contexts, mechanisms of action, and outcomes of sustained and emerging shared care schemes.

**Results:**

Five key themes relating to successful implementation of shared care were identified as (1) clinician-led solutions, (2) redistributing teams, (3) building inter-disciplinary trust, (4) using evidence for buy-in, and (5) standardized care protocols. Scalability was found to be supported by (6) financial incentives, (7) integrated information systems, (8) local governance, and (9) a need for evidence of longer-term health and economic benefits.

**Conclusions:**

The themes and program theories presented in this paper should be considered when testing and scaling shared eye care schemes to optimize benefits and promote sustainability.

## Introduction

As the prevalence of chronic eye diseases rises [1] there is a growing demand for eye care professionals to provide examinations for detection and monitoring of disease, and improve access to interventions. Budget and workforce shortages have challenged the ability of health systems to meet demand and provide accessible and timely sight-saving treatments [2].

To improve access, multiple health systems have introduced task-sharing models of care (known as “shared care” or “collaborative care”) to manage patients with chronic eye diseases. In shared care, traditional ophthalmologist-led tasks are shared with non-medical teams [3]. Often they involve standardized eye examinations being conducted by an optometrist, nurse, or technician who partner with an ophthalmologist to inform a patient’s diagnosis and management [4–6]. However, the mode of delivery and level of task-sharing (including clinical decision-making) varies widely. For example, there are referral refinement schemes whereby community-based optometrists support referral triage [7–9]; hospital-based optometrist-led/nurse-led schemes [5], or community-based clinics whereby optometrists examine patients with ophthalmologist oversight, either direct supervision [10] or virtual review [5, 11, 12].

Extensive evaluations of various modalities—both as pilots and ongoing schemes—have demonstrated that shared care is efficient, safe, and effective. Various schemes have improved access to specialist care and increased hospital capacity [5, 10, 12, 13], reduced service duplication (i.e., diagnostic imaging) [8], improved referral targeting [7, 9] and reduced service costs [10, 12, 14]. Multiple studies have demonstrated that shared care providers who make independent clinical decisions can achieve high levels of agreement with ophthalmologists [7, 8, 10, 12, 13], and deliver reliable clinical assessments [8].

While these benefits are well-documented, fewer studies have investigated the ways these schemes are implemented, the transition from piloting to standard care, or adoption in new settings. This is useful to inform implementation and refinement in new settings, or support scaling-up of existing schemes. The realist theoretical framework can help uncover these program complexities, and identify how different circumstances generate different outcomes [15, 16]. Realist evaluations aim to understand what works, for whom, in what circumstances, to what extent, how, and why [16]. A key assumption is that programs are underpinned by theories which explain the mechanism of change (generative causation) [15].

This study aims to understand the factors which support or impede implementation and scalability of shared care programs in the United Kingdom (UK), Finland and Australia. Using a realist approach [16] the contexts, mechanisms and outcomes of existing and emerging shared care schemes will be identified to inform program theories which can support broader implementation and scalability.

## Methods

### Setting and design

Semi-structured interviews were conducted between October 2018 and February 2020 with key health system stakeholders in the UK, Finland, and Australia. These countries were chosen because they had (1) emerging (Australia), sustained or mature shared care models of care (Finland and UK), and (2) similar health systems (universal health coverage, with public ophthalmology services being free for patients (UK, Australia) or subsidized by regulated co-payments (Finland)).

### Subjects

Participants were purposely sampled from various levels of the health system to capture broad perspectives [17]; and because of their (direct or indirect) involvement in the implementation, adaptations, and ongoing delivery of services. Recruitment included:

(i) Clinicians: ophthalmologists, optometrists, nurses, ophthalmic technicians;
(ii) Managers and administrators: service managers, nurse/optometrist managers; quality improvement and data managers, departments, and hospital leads;
(iii) Governance: regulatory or peak clinical organizations; policymakers.

Prior to the interviews, participants were given an invitation (email or in-person), study information, and signed consent forms. Initial sampling targeted clinicians, often ophthalmologists or optometrists, with introductions made through professional networks of the study investigators. Several participants were sought for their expertise or involvement in formally evaluated and published shared care schemes. Additional stakeholders were nominated by enrolled participants during interviews, either by request of the study team or prompted by the participant. Interviews occurred initially in Finland, followed by the UK, and Australia.

### Data collection and analysis

A semi-structured interview schedule (Supplementary File 1) was adapted using Pawson and Tilley’s “Would it work here?” [18]. Interviews were conducted by three investigators (BF, BA, HL) either in person or by phone for 30 min to 1.5 h. During interviews, prompts informed by published shared care evaluations acted as “program theories” that were tested and validated by participants. The interviewers repeated emerging themes to the participant to allow opportunity for validation of emerging program theories through “theory gleaning” [17]. All interviews included questions regarding unintended outcomes. Interviews were audio-recorded and notes taken. Audio-files were transcribed verbatim by a professional transcription company or one of the investigators (BF) and checked for accuracy prior to analysis. Several participants were invited to review or respond to selected quotes and themes for further theory refinement [17].

Coding and analyses were conducted using NVIVO (12, QSR International 2018). Transcripts were reviewed iteratively, as available. An initial coding framework of 21 codes was derived from the themes and six program theories reported in Baker et al.’s Systematic realist review [6], and were validated using deductive reasoning. The initial coding framework was used by two investigators (BF, BA) to duplicate code four [4] interviews, representing different stakeholder groups from the UK and Finland. An additional 16 codes were identified from the interviews using inductive reasoning. Three investigators (BA, BF, HL) discussed the coding and discrepancies, and came to a consensus on a final coding framework. Preliminary themes and relationships between codes was also recorded. One investigator (BF) used the final coding framework to analyze the remaining 17 transcripts, adding 2 additional codes based on emerging ideas. Interviews involving an investigator as a participant were coded by a second investigator (BA) for rigor.

### Realist evaluation framework

Emerging themes and selected quotes relating to the implementation and scalability of shared care models were identified by one investigator (BF) and presented to the remainder of the study team (LK, BA, AW, HL) at iterative stages for discussion and further refinement. Once themes were established, coding and quotes were reviewed in-depth to generate the contexts-mechanisms-outcomes (C-M-O) configurations for each theme.

### Research team and reflexivity

The research team was multidisciplinary with expertise in ophthalmology (AW), optometry (LK), health economics (HL, BA, BF), public health (all), health administration (BA, BF) and qualitative research (HL, BF, LK). Two members of the study team were considered key stakeholders because they were directly involved in the delivery of a shared care pilot in Australia (AW, BF) and the UK (AW) and were invited to participate. To minimize any bias of being stakeholder-investigators, these interviews were conducted by two external investigators (BA, HL). Four members of the study team had also been involved in evaluations of an emerging collaborative (shared) care model in Australia (BF, BA, LK, AW) [10, 12].

## Results

### Participants

Twenty-one interviews were conducted (Finland—9; UK—5; Australia—7) with participants representing multiple levels of the health system (Table 1). Most participants were clinicians (ophthalmologists, nurses, or optometrists) typically with dual responsibility in management, governance, education, or information technology. Many had initiated shared care, with some having 30-years’ experience; while others learned by participation in ongoing schemes. Several participants had been involved in more than one scheme, including movement across care settings and countries.

**Table 1:** Characteristics of participants and shared care schemes.

Country	Participant type and number represented	Health care and program settings	Eye care funding mechanisms	Design of schemes and chronic eye diseases covered
Finland	Technician (1); Manager/administration, including quality improvement, nurse managers (3); Ophthalmologist, including Chief of Hospital (2); Nurse (1); Optometrist (1); Hospital Director (1)	1x Eye hospital -Tay’s Eye hospital	Municipality/government funded: -Ophthalmologists, nurses and optometrists are employed through the hospital -Patients pay a small co-payment for hospital services (e.g., 30 Euros)	-Nurse-led, with independent decision-making for treatment (glaucoma, AMD) -Nurse-led with ophthalmologist virtual review (glaucoma) -Optometrist-led pre-op assessment (cataract) -Technician led screening (diabetic retinopathy)
United Kingdom	Ophthalmologist, including Heads of Department and scheme Directors (4); Optometrist includes scheme administration/management (1)	3x hospitals -Cambridge Addenbrookes Hospital/ hospital and community optometrists -Bristol Eye Hospital/hospital and community optometrists -Moorfield’s Eye Hospital/hospital optometrists	National Health Service (NHS) Funded: -Ophthalmologists and hospital-based optometrists are employed through hospitals -Community-based optometrists work privately or through University clinics and are paid a fee-for-service from NHS, or in some settings an additional incentive is paid through Hospital Trusts -Patients receive services free of charge	-Optometrist-led referral refinement (cataract, glaucoma) -Optometrist-led community-based clinics with ophthalmologist virtual review (glaucoma) -Optometrist-led hospital/outreach clinics with ophthalmologist direct oversight or virtual review (Cataract, glaucoma, AMD)
Australia	Ophthalmologist, including Heads of Department (3); Policy (1); Optometrist (2); Manager/administrator (1)	3x Hospital -Royal Victorian Eye and Ear/community optometrists -Westmead Hospital Eye Clinic/community optometrists -Prince of Wales Hospital/Centre for Eye Health optometrists -Private ophthalmologists	Government funded (mix of State and federally funded services): -Fee-for-service can be claimed from Medicare (i.e., retinal photography, visual fields, and patient exam) but are used differently depending on the scheme -For hospital-led schemes: ophthalmologists are employed through hospitals, and optometrists are employed independently or through not for profit organizations. Services are free of charge for patients For informal private partnerships: optometrists and ophthalmologists are employed independently, and patients may make co-payments	-Optometrist-led community-based clinics with ophthalmologist direct oversight (cataract, AMD, glaucoma, diabetic retinopathy) Optometrist-led community-based clinics with independent decision-making and ophthalmologist virtual review (glaucoma, diabetic retinopathy) -informal community-based partnerships between private ophthalmology/optometry (variety of diseases)

### Types of shared care

The design, funding mechanisms, and the types of chronic eye diseases examined differed across the settings (Table 1). These schemes included hospital-based, hospital-outreach, or community-based clinics; national screening programs; and informal private partnerships. In Finland and the UK, some had tiered approaches whereby multiple schemes were implemented in one hospital. Shared care was mostly implemented as a formal program and funded publicly through the national health insurance, either through direct employment of staff (e.g., hospital clinicians), or reimbursement using a fee-for-service (e.g., community optometrists). Often patients would receive eye care with no or little co-payment. Australia also had reports of informal arrangements between independent optometrists and ophthalmologists, where funding would come from a mix of patient out-of-pocket (co-payment) fees and government reimbursements.

### Themes

Nine themes relating to the implementation (*n* = 5) and scalability (*n* = 4) of shared care models were derived. Key illustrative quotes for themes are presented in Table 2. The realist context-mechanisms-outcome configurations which inform the program theories are presented in Table 3.

### Implementation themes

#### Access blocks, safety concerns, and clinician driven solutions

This depicted the rationale for initiating the innovation (shared care), and how these models gained traction. Across settings, change was often initiated by clinicians, usually ophthalmologists, but also by nurses, optometrists, and an endocrinologist. Generally, ophthalmologists had observed negative clinical outcomes (vision loss/blindness) in their patients occurring as a direct consequence of being unable to access overburdened ophthalmology clinics. In several settings, clinical safety was the catalyst for change, prompting administrators to look toward clinicians for solutions. (Table 2, quote 1a)

#### Redistribution of Health Care resources, to optimize skill sets, with minimal investment

Redistribution of existing healthcare resources by reorganizing (and upskilling) multi-disciplinary teams through task-sharing arrangements promoted efficiency. For example, fundus photographs taken by technicians, clinical decisions by nurses/optometrists, and support staff transferring patient information. Ophthalmologists generally provided supervision through direct oversight or virtual (store-and-forward) review. Ophthalmologists spent less patient-facing time with the lower risk patients, meaning releasing them to spend more time with complex and advanced patients. Participants often reported that existing resources were rearranged, rather than increased. (Table 2 quote 2b)

#### Interdisciplinary Trust needed to shift clinical responsibility

Motivation and trust were needed to build inter-disciplinary relationships to shift the clinical responsibility from the ophthalmologists to nurses or optometrists. The C-M-O configurations presented in Table 3 demonstrate the various ways that trust was established. Examples include formal training or more organically through daily interactions. Trusting relationships were thought to lead to better clinical decision-making, with a reinforcing effect whereby improved decision-making meant ophthalmologists were more willing to release responsibility. (Table 2, quote 3c)

#### Generating buy-in from decision makers to sustain models of care

Services demonstrated that these models of care were effective, which in turn facilitated teams to get buy-in from decision makers. This included generating evidence on service efficiency, safety, productivity, and acceptability. Participants reported that additional change management could help the transition from pilots to sustained care pathways. For example, regular communication with stakeholders to reinforce the rationale and benefits. (Table 2, quote 4d)

#### Standardized care as equitable care

The use of standardized protocols/processes, care pathways, and proformas streamlined care. This ensured that patients were being managed at a clinically appropriate level. For example, ophthalmologists saw complex and advanced patients, while less-complex patients were assessed by optometrists or nurses. Standardized care was believed to give all patients an equal opportunity to care by removing barriers to access, such as cost or long wait times.

### Scalability themes

#### Health care investment to incentivise and motivate

Incentives are required to motivate and encourage providers to participate and sustain the schemes. Financial incentives were essential to recognize the investments of time, infrastructure, and effort (clinical and administrative) required to deliver services. In the UK and Finland, financial incentives included fee-for-service for community providers (by hospitals), or higher duty payments. Incentives were often justified by the cost-savings accrued through task-sharing. In Australia, the financial model was not clearly developed, and lack of financial incentives available to participating clinicians was a major barrier for sustainability. Participants recognized that funding should be allocated by health departments, and recommended to extend the existing Medicare Benefits Schedule items (telehealth or integrated care) to cover store-and-forward review, or by hospitals introducing an incentive payment for participating private providers. (Table 2, quotes 6f–g)

#### Systems to integrate care and link providers

Health systems need to invest in information technology systems which can transfer patient information between multiple providers. Across settings, there was frustration with current systems which were described as inefficient and clunky. Specific issues were the use of multiple programs and log-ins, and information being transferred via paper and scanned documents. Participants felt that investment in integrated information systems was important for scalability as it could increase efficiency and support continuity of care. Investment in information technology should also consider systems which incorporate data analytics for improved quality control and monitoring of longer-term patient outcomes. (Table 2, quote 7h)

#### Localised governance and monitoring supports quality care

Governance structures and quality assurance mechanisms were required for ongoing program delivery. This covers legal and regulatory issues, program administration, membership or accreditation, ongoing safety and quality monitoring, and development of policy and guidelines. Formal governance processes were necessary when patient volume increased or when models were scaled to include more partners. There were some examples of governance structures which were guided by national clinical guidelines, however more often governance and processes were determined locally. (Table 2, quotes 8i–k)

#### A shift to find evidence of longer-term health and economic outcomes

There is a need to generate evidence of the longer-term health and economic outcomes of these models. Current evidence generally assumed that improved capacity and access leads to better health outcomes. However, longer-term outcomes are not yet understood. Focus should shift to the impact on vision loss and a more wholistic perspective of economic outcomes when patient volume increases; including impacts on access to other eye care services, and health outcomes for patients with more advanced disease, or ocular co-morbidities. (Table 2, quote 9l)

**Table 2:** Themes and illustrative quotes for implementation and scalability of shared care for chronic eye diseases in different health care settings.

Themes	Illustrative quotes
1. Access blocks, safety concerns, and clinician driven solutions	a. “it comes down to consultants wanting to change things for the better of the patients… the delay in review of patients with glaucoma… every single one of those cases where someone had a reduction in vision because of delay, was essentially, was called a serious incident… that gets reported to the National Patient Safety Agency, etc. It’s a very, very big, and rightfully so, a big hullabaloo.” (Ophthalmologist/Manager, UK1)
2. Redistribution of Health Care resources, to optimize skill sets, with minimal investment	b. “you don’t necessarily have to have an ophthalmologist at the top of the tree but essentially the greater the clinical risk of blindness the more involved an ophthalmologist needs to be. [Optometrists] outnumber ophthalmologists six to one. So we need them, just from a workflow point of view. They’re not using the most of their training… it’d be really good to get everyone to work at their best capacity.” (Ophthalmologist/Manager, AUS4)
3. Interdisciplinary Trust needed to shift clinical responsibility	c. “Being clinically competent has to be a key skill of the people who we trust to see our patients. We have to have a level of security that, what they’re saying and what they’re seeing is correct and accurate…[and] confidence in the findings because especially with things like pressure, you know, if it’s a nonsense [unreliable/inaccurate intraocular] pressure, that makes a big difference to the clinical management of the patients. We do a lot of training with the community optometrists before they’re left to do their community glaucoma clinics, and I guess that trust that you mention, and that confidence builds up during that training period.” (Hospital Optometrist/Manager, UK4)
4. Generating buy-in from decision makers to sustain models of care	d. “it started off as a pilot scheme, which then just never stopped. We were able to demonstrate that of our new patients, we could discharge 50% of them before they even got to the hospital and that was very powerful in buying-in….I think everyone realizes that it’s here to stay now.” (Ophthalmologist, UK2)
5. Standardized Care as equitable care	e. “Not everybody who gets sent to that kind of system needs to be in that system in the first place….We tended to see a lot of is people who fall through the cracks because they can’t afford care and can’t afford to wait for care. So, they end up not going anywhere. Some of those cases were actually really quite complex cases that you would need to be seen by an ophthalmologist, but was basically not seeing anybody.” (Optometrist, AUS5)
6. Health care investment to incentivise and motivate	f. “It’s part of the work that doctors did before…it was such an important job, and it made our doctors have more time to do other things when the nurses took part of their job. So that was the reason that they [nurses] got more salary… it’s about 10%, and the volume of patients is a lot more.” (Hospital Director, FIN9) g. “…all these things require some sort of financial support to get them off the ground, get them working and for people to be remunerated appropriately so they stick with it and it becomes something that’s sustainable.” (Optometrist, AUS5)
7. Systems to integrate care and link providers	h. “it’s making sure that there’s clear communication but it’s obviously safe communication, it’s two-way, and it talks to all the systems as well. We don’t need another standalone system, we need something that’s integrated, because nobody has time to write the same thing twice. We need things to be able to go backwards and forwards in a safe, reliable manner, and easily updated when needed to be” (Policy, AUS2)
8. Localised governance and monitoring supports quality care	i. “…before I did anything, I contacted the Finnish Health Authority and explained what we’re planning to do, and ask them if is this okay. And they say, well I’m responsible for everything and if I think this is fine, it’s fine. So, I first wanted to make sure that it’s legal.” (Ophthalmologist/Chief Executive, FIN3) j. “I set up the policies and protocols and sought out the service level agreements through our Commissioning Department here at the hospital… we have named an accredited optometrist at each practise… People who failed to attend the annual accreditation meeting, or if they don’t respond to direct feedback about poor referrals or something like that then we’ll usually try to find out why. Usually because they’ve just left the area or something like that… but yeah, we have terminated people from the scheme in the past” (Hospital Optometrist/Manager, UK4) k. “you need to account for local variations… some overarching regulation, particularly for your more established diseases where you’ve got a really strong evidence base behind how they should be managed… You will need some models of care to evolve more organically or on a local level.” (Optometrist, AUS6)
9. A shift to find evidence of longer-term health and economic outcomes	l. “The big four eye diseases account 70% of our patients costs and visits. Then we had the whole package… it’s very, very interesting to see because if we are spending more money on AMD and if our budget is not increasing to the same extent, we have to do something else somewhere in some other disease… Otherwise it’s very hard to prove that what you are doing makes sense even if you could show that it saves cost here, but if it incurs costs somewhere else.” (Ophthalmologist, Chief Executive FIN3)

**Table 3:** Context, mechanism, and outcome configurations for implementation and scalability of shared care for chronic eye diseases in different health care settings.

	Contexts	+	Mechanism	=	Outcomes
Theme one: Access blocks, safety concerns, and clinician driven solutions	Overall: Patient numbers grow rapidly; limited financial resources; shortages of ophthalmology workforce; belief that situation will become even more unmanageable	+	Overall (i) Clinicians identified solutions to improve patient outcomes (ii) Wait-lists and adverse clinical outcomes data supports administrator buy-in	=	Overall: Administration and policy makers relied on ophthalmologist expertise to lead the innovation; Ophthalmologists remained responsible for the patient’s care through optometrist/nurse oversight and training; uncertainty and resistance from some ophthalmologists, requiring additional engagement, but eased when the model benefits shown. Ophthalmologists were confident the models could identify patients who most needed ophthalmologist care. They were essential to manage large volumes of chronic eye disease. Patient numbers were higher than expected (unexpected outcome)
	Finland: National care guidelines mandate maximum wait times; outsourcing to private services to meet demand	+	Finland (i) Negotiation with health services to ensure clinical leadership including budget and staffing		
	United Kingdom: National clinical guidelines prompted more ophthalmic referrals; Ophthalmologists saw vision loss/ blindness because patients could not access services; lack of physical space to expand	+	United Kingdom + Australia (ii) ‘Catalyst moments’ after serious adverse incidents were reported to safety agencies and escalated in health departments; business cases prepared by ophthalmologists; pilots demonstrated models were safe and effective to get support from administrators		
	Australia: Ophthalmologists saw vision loss/ blindness because patients could not access services				
Theme 2: Redistribution of Health Care resources, to optimize skill sets, with minimal investment	Overall: Hospital budgets were limited and could not be extended to meet the growth of chronic eye diseases; limited resources including ophthalmologist workforce, equipment, and space	+	Overall: (i) Increase workforce through task-sharing and upskilling to manage the lower-risk patients (ii) Ophthalmologist level skill for advanced/complex patients (iii) Greater productivity (i.e., patient volume) (iv) Little additional investment. Policymakers were attracted to models which met the service needs within existing resources.	=	Overall: Schemes were deemed acceptable if the same level of care was delivered and no harm was done; Quality of care for high-risk patients would improve through more time with an ophthalmologist United Kingdom + Australia Clinicians felt increased access and capacity were of high importance, even if the models of care had no overall cost-saving or were cost neutral (unexpected outcome)
		+	Finland (i) Expanded through nurses, technicians, and optometrists (ii) Increased resource use was not believed to guarantee better health outcomes		
		+	United Kingdom (i) Expanded through community/hospital optometrists and additional training (ii) New equipment purchased as needed (iii) Pilot funding	=	United Kingdom + Australia Clinicians felt increased access and capacity were of high importance, even if the models of care had no overall cost-saving or were cost neutral (unexpected outcome)
		+	Australia (i) Expanded through community optometrists with existing clinical skills (ii) Pilot funding and non-government partnerships		
Theme 3: Interdisciplinary Trust needed to shift clinical responsibility	Overall: Traditionally ophthalmologists held power and responsibility for patient outcomes; Initially assessments were being sent to ophthalmologists for checking. The definition of shared care, including the amount of oversite required (e.g., face to face, virtual clinics) differed across settings, even within the same country and professional groups	+	Overall (i) Multidisciplinary teams had a common goal of improved care (ii) Training and feedback were introduced (iii) Developing relationships and navigating personalities organically (iv) Select motivated partners, (i.e., willing to change or local knowledge)	=	Overall: Trust developed organically through daily interactions and ongoing communication; improved confidence in decision-making by the nurses and optometrists and high levels of clinical agreement; improved decision-making meant ophthalmologists released more responsibility; optometrists and nurses were empowered through increased skills and scope of practise; collective leadership and mutual respect and learning, and that benefits extended beyond the scheme into other aspects of clinical care (unexpected outcome).
		+	Finland (i) Ophthalmologists provided hands-on training of nurses.		
		+	United Kingdom (i) National level formal training for optometrists (Enhanced Optometry Scheme) (ii) Ongoing accreditation, feedback, and training organized locally		
		+	Australia (i) Some ophthalmologists believed that optometrists had adequate training to make clinical decisions using only protocols, virtual review, and regular informal feedback (ii) Some ophthalmologists provided direct oversight, and optometrists completed formal training (i.e., modules, didactic)	=	Australia Some optometrists said additional training was unnecessary and wanted more independent responsibility (unexpected outcome).
Theme 4: Generating buy-in from decision makers to sustain models of care	Overall: Traditional medical model (ophthalmologists-led) being transferred to optometry/nursing was a radical change; clinical teams developed business cases; overarching goal to allocate appropriate care level and transfer low risk from an ophthalmologist to optometrist/nurse.	+	Overall (i) Published research and informal measures to demonstrate effect (ii) Increased patient volume (iii) Cost measures varied with implementation modality (iv) Patient satisfaction was not directly measured but could use feedback surveys or patient complaints	=	Overall: Evidence generates buy-in from decision makers, and enables models of care to continue and expand; additional change management needed to convince clinical teams that this was business as usual
	Finland: Overall responsibility of staff and budgets given to the Head ophthalmologist.	+	Finland (i) Productivity measured through allocation of resources and outputs for all eye care services (one piece of pie)		
	United Kingdom + Australia: Policy and decision makers requested evidence through piloting, implementation, and modeling data; pilot studies were often funded through government or hospital grants.	+	United Kingdom + Australia (i) Wait times (getting timely treatment) (ii) Patients avoiding hospital (Referral refinement schemes) (iii) Clinical safety and clinician agreement (Virtual review) (iv) Adherence to clinical protocols or national benchmarks (v) Cost measures compare shared care models to traditional care, and informally cited staff time/resources	=	United Kingdom + Australia Pilots naturally evolve into standard care, even if no desired effects (unexpected outcome); believed to be cost-effective (support from administrators), but some uncertainty if actually cost-effective (unexpected outcome); minimal cost increase to improve capacity and wait times was acceptable (unexpected outcome)
Theme 5: Standardized care as equitable care	Overall: Barriers to accessing care—high patient volume and wait times—meant no individual care Australia: Affordability (high costs). Large volume—patients did not get individual care	+	Overall: (i) Differentiates those needing specialist care (ophthalmologist) from lower levels of care (optometrists and nurses). (ii) Protocols, proformas, processes, and pathways were used	=	Overall: More patients accessing service and receiving appropriate care (more equitable). Low risk patients being managed well by nurses/optometrist (with some ophthalmologist oversight) Not suited for all patients (e.g., ocular co-morbidities, rapid ophthalmic changes, cognitive and mobility issues, or medical co-morbidities). Several settings introduced tiered models.
		+	Finland (i) Consistency through returning to the same clinic for examinations (ii) Virtual care allowed multiple patients to receive tests at the same time (for example, visual fields).		
	Australia: Affordability (high costs). Large volume—patients did not get individual care		United Kingdom and Australia (i) Streamlines referral and triage processes (ii) Same equipment to transfer information between providers		
Theme 6: Health care investment to incentivise and motivate	Overall: Reliance on clinicians with special interest or extending the tasks of existing teams.	+	Overall (i) Time, effort, and money were needed to sustain the models.	=	Overall: Risk that models would not be sustained without financing
	Finland: Schemes had been in place for 5–10 years, but not all staff wanted to be involved	+	Finland (i) Higher duty salaries paid to nursing staff trained in shared care (virtual clinics) to recognize time/skill. Rationale that tasks previously assigned to ophthalmologists.	=	Finland and United Kingdom: Incentives motivated providers to participate, to cover time or invest in skill development. Resulted in higher patient volume and identification of more disease.
	United Kingdom: Pilot funding was often temporary; but some schemes had been in place for up to 30 years; various financial models utilized.	+	United Kingdom (i) National health Service (NHS) funding schemes (GOS/EOS) supported some components of shared care. (ii) One hospital paid fee-for-service to local accredited optometrists for cataract and glaucoma assessments. The fee was from hospital allocated funding for the service		
	Australia: Temporary pilot funding; no established funding; Relied on good will of clinicians, informal partnerships in rural areas where ophthalmologists were scarce and time poor.	+	Australia (i) Current funding (e.g., Medicare) does not support activities (e.g., virtual review) or not billable by all providers (ii) Recommended current Medicare items be extended for shared care (iii) Savings of task-shifting could be reallocated to finance incentives	=	Australia: Lack of financing is a major barrier for scalability. Without clear funding, it was not worth providers giving up practice time. Also needs commitment from health systems that timely and affordable access to treatment/surgery is available when disease is detected.
Theme 7: Systems to integrate care and link providers	Overall: Patient information needs to be shared between multiple clinicians. Current systems not built for shared care, deemed inefficient, clunky, patchy, or ad hoc, and high administrative burden. Systems could not handle large volumes of patients.	+	Overall: (i) Investment in IT solutions to support shared care was seen as an urgent priority, but funding for IT systems was harder to negotiate with administrators (ii) Ideal systems have free flow and two-way communication of patient information (iii) Transfer of both clinical and administrative booking information (iv) Secure systems that comply with privacy and confidentiality requirements	=	Overall: Integrated systems would support quality care (patients do not slip through the gaps), care continuity, and feedback between providers
	Finland: Multiple IT systems with separate log-ins.	+	(i) Enhanced IT systems could be used to track health outcomes	=	Finland: IT breakdowns were stressful, as it slowed down care in fully booked clinics (unexpected outcome). A new integrated database would enable real-time reporting of patient health outcomes.
	United Kingdom and Australia: Optometrists and hospitals use different equipment (i.e., clinical technology). Some programs used paper files, emails, and scanned documents; other programs established remote logins to hospital programs for community providers.			=	United Kingdom: Purpose-built centralized IT systems (e.g., EPIC, New Medica) for store-and-forward care. Can be accessed by any licensed provider (despite locations). offer feedback and continuity.
		+	Australia (i) New systems need integration with health care systems, not stand alone	=	Australia: Patient-centered systems, adaption of existing programs for shared care (e.g., My Health Record). Store-and-forward systems would allow patients in rural/ remote areas to be seen closer to home, and ophthalmologists (in any location) review batch records.
Theme 8: Localised governance and monitoring supports quality care	Overall: Monitoring ensures patient safety and quality of care is maintained; the volume of patients increased more quickly than expected.	+	Overall: Governance processes and quality assurance checks were included to all settings, but implementation varied across settings.	=	Overall: Local governance at the hospital level. National guidelines supported uptake in new areas. Quality checks maintain care standards and introduce improvement.
	Finland: Head ophthalmologist sought advice to ensure the models complied with legal and regulatory requirements; no existing local or national guidelines; local governance.	+	Finland (i) Ongoing audits for monitoring/reporting (ii) Regular team communication to review data and quality improvement	=	Finland: Ophthalmologists lobbied health departments to get patient prioritization models in national strategies for broader uptake.
	United Kingdom: No national guidelines available before the pilot/s started. Increase in patient volume, some hospitals needed to quickly engage more partners. Schemes implemented at local level.	+	United Kingdom (i) Agreements/contracts with key performance indicators signed by hospital and community providers. (ii) Accreditation and continued professional development iii) Formal and informal feedback sessions (iii) Regular audits	=	United Kingdom: After initial pilots, national guidelines were written, promoting shared care as recommended practice in other hospitals. Decisions to implement remain local level (i.e., Trust/Commissioners)
	Australia: Advice sought to ensure the shared care models complied with legal and regulatory requirements. Guidelines for shared care were prepared by national clinical peak bodies and informed from evidence in overseas models. Examples of programs led by public hospital with community providers, and also informal partnerships between private providers in both rural and metropolitan locations.	+	Australia (i) Agreements between hospital and community providers (ii) Formal and informal feedback sessions (iii) Informal partnerships developed organically through local connection and did not have formal agreements or protocols (iii) In rural/remote Australia formal governance could be enacted by organizations with wide geographical coverage (e.g., Primary Health Networks, Glaucoma Australia, professional groups).	=	*Australia:* Governance and implementation approaches are flexible to suit the context (e.g., rural areas, hospital-led, informal partnerships).
Theme 9: Evidence of the longer-term health benefits	Overall: Urgency to implement solutions to increase capacity. Published evidence is from pilot/short-term follow up. This shows patients can access care faster, high-risk patients are identified for more intensive ophthalmic management. Lack of research/surveillance funds, and poor data quality (due to non-integrated data systems) means longer-term health and economic outcomes are less often evaluated. Long-term health outcomes can also be difficult to measure due to the variable/progressive nature of chronic eye diseases. Patient experiences not always captured.	+	Overall: Overarching assumption that improved access and frequent monitoring will identify at risk patients and offer timely intervention—ultimately resulting in better health outcomes.	=	Overall: There needs to be more evidence generated on the impacts of shared care on health and economic outcomes relating to the prevention of vision loss and blindness. As well as the impacts for other eye conditions.
		+	Finland: (i) Focus is on identifying those at highest need for ophthalmologist intervention, while monitoring lower-level disease		
		+	United Kingdom and Australia (i) Acceptance that improved access means clinicians can deliver earlier intervention to halt progression of disease. (ii) Anecdotal reports of changes to the hospital’s clinical case-mix following implementation, thus long-term outcomes for advanced disease could be impacted.		

## Discussion

Informed by broad health system stakeholder experiences, this study has drawn evidence from shared eye care interventions at different stages of maturity and within various health system contexts to build an understanding of what factors influence implementation and transition of pilots into scaled and sustained programs. The use of the realist approach meant testing and validation of program theories could evolve our knowledge of how these program work [16]. The implementation themes presented in this paper were reported consistently across settings, informing overarching theories to explain why these programs work. Within complex health systems, it has been suggested that innovations with obvious effect will be more readily adopted [19]. In this study it was universally recognized that the impetus for change was the high demand for eye care due to the growing population of patients with chronic eye diseases and adverse vision outcomes due to delayed access. This led to health system administrators to seek sustainable and acceptable solutions (task-sharing) to increase capacity and improve access. However, the implementation of these solutions varied greatly across and within countries. By adopting a realist approach, this study has been able to establish the context-mechanism-outcome configurations (Tables 1 and 3) to provide insight into what works and how this is achieved across contexts [16].

When it came to scalability, these themes were informed by stakeholder’s experiences of problems/solutions; not only from sustained models of care but also the perceived barriers for broader uptake in systems where these schemes are still emerging. Participants in all settings strongly believed that health system investment was needed to scale services, notably through remuneration and information technology models. These investments would ensure longevity of outcomes and productivity gains.

This study built on theories reported by Baker et al. [6] which describe the success and failures of similar shared eye care in the UK. Several theories were validated. For example, participants across settings validated the effectiveness of task-sharing (Theme 2; Baker et al. Theory 1), the importance of genuine local partnerships for implementation (Theme 3 and 8; Baker et al. Theory 2), and the benefits of standardizing care (Theme 5; Baker et al. Theory 3). However, Baker et al.’s [6] program theories were founded on studies which report mostly on outcomes and effectiveness of interventions, and were less focussed on implementation processes and contextual factors (Baker et al. Theory 6—Barriers and enablers). Thus, in this study, an important methodological component was inductive analysis to refine and generate new program theories to explain aspects of program implementation. In this study, clinician leadership (Theme 1) and interdisciplinary trust (Theme 3) were mechanisms needed to effectively facilitate task-sharing and shifting of responsibility as reported in Baker et al.’s Theory 1.

Task-sharing in health care is not new and has been used widely in other disciplines to address some of the same obstacles seen in eye care; and can be useful in low income countries where health care resources are scarce [20]. In Australian and UK settings, a prominent example of scaled shared care are antenatal programs which involve GPs (or midwives) and obstetricians. These programs are standard practice in most public hospitals and national clinical guidelines ensure fidelity remains. However, these programs can be adapted locally to provide responsive care (e.g., tiered approaches such as midwifery group practice or GP-obstetrician programs [21].) In another example, Kemp et al. [22] suggest that scaling (national and international) of a maternal and child health program was achieved by allowing local adaptations while maintaining program fidelity. They liken this to baking a basic cake (core components) and adding recipe variations (local adaptions) to adjust flavor.

Similar core components exist in shared care initiatives used in eye care and other health disciplines. This includes supervision, training or upskilling workforce [20, 22]; well-defined scope of practice [20, 23]; protocols [20, 22, 23]; and service integration [21, 22, 24]. Information technology is recognized as an essential component for team communication [23, 24]. Within eye care, large-scale diabetic retinopathy screening programs in the UK rely on teleophthalmology (store-and-forward) to transfer information across providers [25]. In India, electronic medical records that interlace through a three-tiered eyecare network (primary care, secondary clinics and tertiary eye hospitals) are able to capture data and imaging for large populations over time. Furthermore, digitization of health information allows real-time analytics which can support quality control and research to measure longer-term health outcomes [26]. In this current study, several participants reported investment into local IT systems. One UK example is a web-based patient record (New Medica) which has facilitated almost 25,000 virtual consultations across multiple clinics. By embedding automated quality checks and real-time feedback for optometrists, Wright et al. were able to demonstrate improvements in patient care [27]. However, Sim et al. [25] report that lack of integration with a nationally accessible electronic health system is a known barrier to uptake of teleophthalmology in the UK. In our study, participants were unsatisfied with current IT systems due to lack of integration and inability to extract meaningful data. Thus, longer-term health and economic outcomes cannot be efficiently monitored; and services were unable to make informed decisions on how to allocate resources to achieve the best outcomes [28].

Information technology is not the only challenge. Resistance to change from both organizations and providers is regularly cited as a key concern across disciplines for the successful uptake of shared care [20, 22, 25]. Participants in this study suggested change management strategies were needed, including regular communication and engagement for teams, external clinicians, and stakeholders; motivation (and incentives) for providers; and involving the right people, such as clinicians, to lead the change. Other studies recommend that effective leadership [20, 25] or local champions are needed to gain “buy-in” [22] and drive change. However, team culture and power imbalances should be addressed to support uptake [19, 24]. In this study, interdisciplinary trust evolved organically through daily interactions and training. One Australian study examining interprofessional relationships of shared eye care providers found that trust is established by regular contact or co-location of providers [23], however in another study too much oversight and scrutiny of optometrists work resulted in diminished levels of trust [29]. Thus, for broader implementation, pre-planning should consider including change management strategies to facilitate adoption.

### Study limitations

This study has several limitations. First, it does not capture the concurrent experiences of piloting and implementation of shared care models, and relied on retrospective accounts from various stakeholders, some of whom had been involved in the initial pilots, program modifications, and ongoing implementation. However, the schemes in Finland and UK were in place for many years which provides valuable insight into sustainability. Second, being a qualitative study, the sample is limited to 21 participants from three countries and does not reflect the experiences of all shared eye care programs, particularly the barriers for programs that have been disbanded when unsuccessful. The context-mechanism-outcomes configurations are based on participant perceptions of events or ability to recall, thus may not cover all aspects of local implementation. However, the sample was purposely selected to reflect a wide range of by experts who have been involved in piloting, program evolution, evaluation and the development of policy, guidelines, and education.

## Conclusions

Using a realist approach, this study has identified nine key factors relating to the implementation and scalability of shared eye care programs in the UK, Finland, and Australia. Overall, implementation is supported by clinician-led solutions, rearranging multidisciplinary teams, building inter-disciplinary trust, generating local buy-in, and using standardized care protocols. Scalability will require investment from broader health systems to support financial incentives to motivate providers and integrated information systems. There was a preference for schemes to be governed locally, allowing for flexible implementation. However, evidence is still needed for longer-term health and economic outcomes when scaled. Shared eye care is necessary to tackle the growing demands for eye care services, and for these programs to be effective, equitable, high quality and sustainable, findings should be systematically addressed.

## Summary

### What was known before

- There is a growing demand for chronic eye care services to detect, monitor, and treat disease.
- Shared care between ophthalmologists and optometrists can increase access for patients, and improve efficiency and cost-savings for health systems.

### What this study adds

- Successful implementation of shared care is enabled with clinician leadership, redistribution of resources, trust, monitoring, and standardized care.
- Scalability of shared care can be supported through incentives, integrated systems, governance, and evidence of longer-term benefits.
- Health system and policy stakeholders perspectives and the realist framework assist in understanding implementation and scalability of shared eye care schemes.
- These findings can guide eye care professionals in planning and expansion of shared eye care in new settings.

### Supplementary information

Supplementary File 1

## Data Availability

Deidentified participant data are available from authors upon reasonable request. Access to any data will require ethical review and approval from the UNSW HREC, and may require investigators to collect additional individual consent from participants for further use of data.
