# Supplementary material for: Implementation and scalability of shared care models for chronic eye disease: a realist assessment informed by health system stakeholders in Finland, the United Kingdom, and Australia
Source: Eye (Lond). 2023 Mar 6;37(14):2934–45. doi: 10.1038/s41433-023-02444-9 (PMC10517111; doi:10.1038/s41433-023-02444-9)
Supplement: Supplementary file 1 [file 41433_2023_2444_MOESM1_ESM.docx]

**Supplementary File (online only)**

**Supplementary File 1: Semi-structured realist interview schedule**

|  | **Existing System (UK and Finland)** | **New System (Australia)** | **Desirability and/or feasibility of changing practice, procedures and context of system B to match those of system A** |
| --- | --- | --- | --- |
| **Icebreaker** | What is your role and how are you involved in shared/collaborative eye care?  What does shared/collaborative eye care look like in [insert country/ hospital]?  What are the major eye diseases covered in shared/collaborative care models in [insert country]? | | |
| **The innovation** | What are the key features of shared/collaborative care models for chronic eye disease in your health service? (Provider/ Manager)  How did shared/collaborative care modify the way routine care was delivered for chronic eye disease patients? (Provider)  How does shared/collaborative care align with/support the goals of broader health service? What is the purpose? (Policy/ Administrator/ Governing bodies) | In your opinion, what features would be essential in a shared/collaborative care model in Australia? (Provider/ manager)  How could shared/collaborative care models change the way that routine eye care is delivered in Australia? (Provider/ Manager/ Policy)  How would shared/collaborative care support the goals of the your health system? (Policy/ Administrator/ governing bodies) | When implemented in [country/ health service] they suggested [feature] was important, what do you think about this in [country] shared/collaborative care models? |
| **The resources** | What kinds of resources are needed to successfully deliver shared/collaborative care (staff time, money, equipment, space, etc) I your hospital/health system?  On an ongoing basis, how many staff are involved in delivery of shared/collaborative care? (provider, manager, governing body)  Can you describe the role of health information technology and other equipment in the success and scalability of model of care? (Provider, manager, policy)  Can you describe how collaborative care services are funded and any infrastructure required? (Manager/ Policy) | What resources are needed for the Australian health system to introduce collaborative care models more widely? (Provider, administrator, policy)  What is needed in health information technology/ equipment/ funding for collaborative care in Australian health systems? (Provider/ Manager)  How should collaborative care services be funded/ any infrastructure required in Australia for expansion of services? (Manager/ Policy) | When implemented in [country/ health system] they use [resources], how did/would this work in [country]? |
| **The people** | Who are the staff involved in shared/collaborative care at your health service? What are their roles? What is their training? (Provider, manager)  From a [policy/ provider/ administrator/ governing body] perspective, what are the characteristics [skills, expertise, experience, training, commitment] of the key players of shared/collaborative care? What did you need to change? How has this improved the implementation at your service?  How has the relationship between optometrists and ophthalmologist been affected from participation in shared/collaborative care? Were there any actions needed to improve this relationship? (Provider, governing body) | What are the salient characteristics of the key actors in system B?  Who are the key players [staff types, organisation, governance] that should be involved in shared/collaborative care in Australia? (Provider/ Management/ Governing)  Is there anything required to make the key players ready for expansion of shared/collaborative care models? | Shared/collaborative care models in [country/ health system] rely on [key players], how does/ would it work in [country]? |
| **Institutional factors** | How do you think external factors [organisational, departments] have influenced the decisions to implement a shared/collaborative care models at your service? Beyond immediate clinics team, what kind of roles were involved? (Provider/ Manager)  Who were the decision makers and change leaders? What was needed to prepare the organisation/ teams for change? (Manager, Administrator, Policy, Governance)  How are partnerships between the hospital and optometrist governed? [e.g. payments, reporting lines, communication] (Manager, Administrator, Governing bodies) | Within the hospital/ health system what external factors [organisational, departments] would influence the adoption and scalability of shared/collaborative care? (Provider, Administrator)  Who are the decision makers and change leaders that need to be involved? (Manager, Administrator, Policy, governance)  How should shared/collaborative care be regulated and governed in Australia? [e.g. payments, reporting, communication] (Policy, Governance) | Shared/collaborative care models in [country/ health system] rely on [external institutional factors], how does/ would it work in [country]? |
| **Environmental factors** | How did the decision makers and policy makers become invested in the model of care? (Provider, Administrator)  Were there any regulatory issues in relation to shared/collaborative care that needed to be overcome before implementation? How did you go about overcoming this? E.g. accountability (Provider, manager policy, governing body) | Are decision makers and policy makers already invested in shared/collaborative care? (Provider, Administrator, Policy)  Are there any regulatory issues that need to be considered before implementation? (Providers, Policy, governing bodies) | Shared/collaborative care models in [country/ health system] relied on [political, legislation] factors for successful implementation, is this needed in [country]? |
| **Measures** | How do you measure the success of the shared/collaborative care models at your [service/ health system]? (Provider, Manager, policy)  What key indicators [baseline, process, outcome] determine success? (Provider, manager, policy)  How do you identify failures or introduce improvements in the program? (Provider, manager) | How would we measure success of shared/collaborative care models in Australia health services? (Provider, Administrator, Policy)  What key indicators [baseline, process, outcome] would determine success? (Provider, manager, policy)  How would you identify failures? How would you introduce improvements? (Provider, administration, policy) | In [country/ health system] success is measured by [indicators], would you use the same measures in [country]? |
| **Procedures** | Can you describe the exact process of shared/collaborative care in [health system]? What was introduced in [health system] to lead to the success of your shared/collaborative care service? (Provider, manager, policy)  How have patient care outcomes changed since shared/collaborative care models were introduced? (Provider, manager)  How do you ensure there is adequate communication between shared/collaborative care providers? Can you provide an example of where this communication works well and when it does not? (Provider, manager) | In [health system] shared/collaborative care involves [process], how would this work in Australia? What would the outcomes be? (Provider, manager, policy, governing body)  How would patient care outcomes change with the introduction of shared/collaborative care? (Provider, policy)  How would communication between providers work in Australia? Can you think of any strengths or weaknesses to this approach? (Provider, policy, governing body) | Studies from [health system] have suggested that standardisation of care and protocol driven care are important, can you describe if/how this would be beneficial to providing care in [health system]? |
| **Outcomes** | What were the key outcomes for [patients/ staff/ health system]? What was the cost to gain these successful outcomes?  (Provider, manager, policy, governing body)  What are some of the changes you see in your own professional practice because of shared/collaborative care? (Provider, governing body)  Were there any unintended changes? What are somethings that are working well and not so well? (All)  Are there any types of patients that you think would benefit most from this service, are there any that would not? (Provider)  In your opinion how appropriate is shared/collaborative care for ongoing management of low risk chronic eye disease patients? If negative: in your view, what would be a more appropriate outcome for this patient? (Provider, governing body) | What do you think will be the key outcomes of shared/collaborative care models for [patients/ staff/ health system]?  (All)  What investment is needed to gain these successful outcomes?  (Provider, manager, policy, governing body)  In your opinion how appropriate is shared/collaborative care for ongoing management of low risk chronic eye disease patients? If negative: in your view, what would be a more appropriate outcome for this patient? (Provider, governing body) | I have read that shared/collaborative care models in [health system] have [outcomes], how do you think this would be a benefit in management of patients? |
